# WITHDRAWN: Supinoxin blocks Small Cell Lung Cancer Progression by Inhibiting Mitochondrial Respiration through the RNA Helicase DDX5

**DOI:** 10.21203/rs.3.rs-4169007/v1

**Published:** 2024-04-15

**Authors:** Subhadeep Das, Matthew P. Russon, Maria P. Zea, Zheng Xing, Sandra Torregrosa-Allen, Heidi E. Cervantes, Haley Ann Harper, Bennett D. Elzey, Elizabeth J. Tran

**Affiliations:** 1 Department of Biochemistry, Purdue University, BCHM A343, 175 S. University Street, West Lafayette, Indiana 47907-2063; 2 Purdue University Institute for Cancer Research, Purdue University, Hansen Life Sciences Research Building, Room 141, 201 S. University Street, West Lafayette, Indiana 47907-2064; 3 Department of Comparative Pathobiology, Purdue University, West Lafayette, IN, USA

## Abstract

The authors have requested that this preprint be removed from Research Square.

